# Giant retroperitoneal dedifferentiated liposarcoma with intraperitoneal extension: A case report

**DOI:** 10.1016/j.radcr.2026.08.028

**Published:** 2026-09-11

**Authors:** Kamal Hlioui, Loubna Messari, Fatma El Mabrouk, Ouijdane Zamani, Tarik Salaheddine, Rachida Saouab, Jamal El Fenni, Meriem Boui

**Affiliations:** Hopital Militaire d'Instruction Mohammed V, Rabat, Morocco

**Keywords:** Retroperitoneal tumors, Dedifferentiated liposarcoma, Malignant fat-containing tumor, Radiologic-pathologic correlation

## Abstract

Retroperitoneal liposarcoma is a rare malignant mesenchymal neoplasm of adipocytic origin that often remains clinically silent until it reaches a considerable size. We report the case of a 66-year-old woman presenting with progressive right lower abdominal pain and a palpable abdominal mass. Initial ultrasonography demonstrated a large heterogeneous subhepatic mass containing both macroscopic fat and solid components with cystic areas. Contrast-enhanced CT and multiparametric MRI further characterized the lesion, revealing a giant retroperitoneal mass with secondary intraperitoneal extension, abundant macroscopic fat, a large enhancing nonlipomatous component, thick septa, necrotic areas, and diffusion restriction, findings highly suggestive of dedifferentiated liposarcoma. Histopathological confirmation was obtained by image-guided core needle biopsy. Owing to the extensive local disease and close relationship with major abdominal vessels, the lesion was initially considered unresectable, and the patient was referred to the multidisciplinary sarcoma team for systemic chemotherapy. We report the case of a giant dedifferentiated retroperitoneal liposarcoma in a 66-year-old woman which highlights the pivotal role of multimodality imaging in the characterization of giant retroperitoneal liposarcomas, enabling accurate preoperative diagnosis, biopsy guidance, and therapeutic planning.

## Introduction

Retroperitoneal liposarcoma is a rare malignant mesenchymal tumor that poses a diagnostic challenge because of its nonspecific clinical presentation and the broad differential diagnosis of fat-containing retroperitoneal masses. We report the case of a giant dedifferentiated retroperitoneal liposarcoma in a 66-year-old woman, highlighting the key contribution of multimodality imaging in lesion characterization, prediction of the histological subtype, and preoperative therapeutic decision-making.

## Case presentation

This is a 66-year-old female with no significant medical history, admitted to the emergency department for localized abdominal pain and a sensation of heaviness in the right iliac fossa. The symptoms had progressively developed over the past few weeks without any other associated functional signs. Abdominal examination revealed a large, palpable and firm mass in the right iliac fossa extending to the flank. The mass was immobile relative to both deep and superficial planes, nonpulsatile, painless, and without any signs of inflammatory skin changes over the area.

**Fig. 1:**
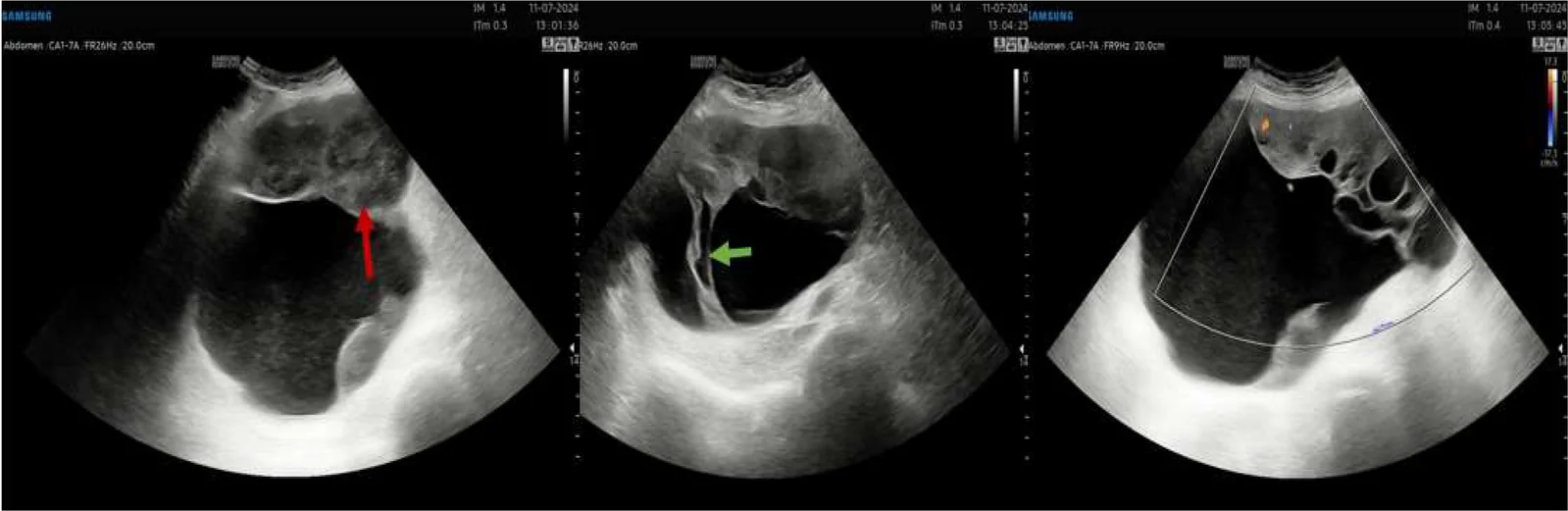
Ultrasound images showing the mass with a fatty component (red arrow), a fluid portion and thickened septa (green arrow).

Given a normal biological profile, an abdominal-pelvic ultrasound was indicated (Fig. 1), which revealed a giant subhepatic mass with both adipose and tissue components containing a cystic portion. The tissue portion showed low vascularization on color Doppler. This mass displaced the liver, intestinal loops, kidneys, and vascular structures, without clear signs of invasion.

**Fig. 2:**
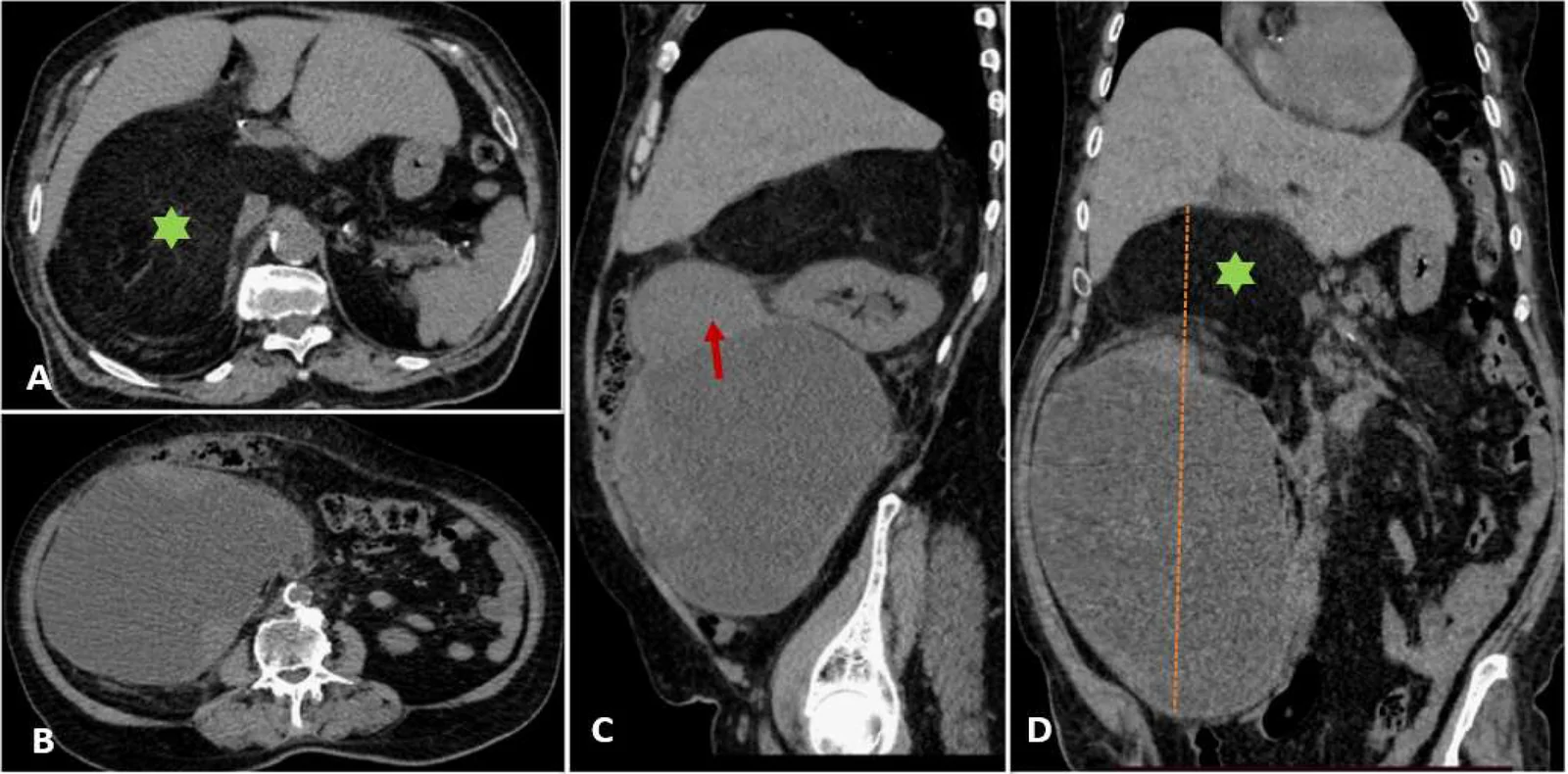
Abdominopelvic CT scan axial view (A and B) with sagital (C) and coronal (D) reconstruction, showing large retroperitoneal extensive mass with a large lipomatous portion (green star), soft tissue component (red arrow), and cystic portion.

In front of the large craniocaudal extent of the lesion (>200 mm) and the need to better define its anatomical origin and extension, abdominopelvic CT scan with contrast enhancement was performed (Fig. 2). CT demonstrated a giant right retroperitoneal abdominopelvic mass arising from the hepatorenal space, with secondary intraperitoneal extension. The lesion showed a heterogeneous composition, combining abundant macroscopic fat with a large nonlipomatous soft-tissue component. The latter contained thick internal septations and showed mild heterogeneous enhancement after contrast administration, with non-enhancing necrotic areas. No calcifications were identified. The mass was associated with marked infiltration of the adjacent right retroperitoneal fat planes, supporting an aggressive retroperitoneal behavior. Good opacification of the vascular structures was observed, with no expansion of the urinary tract.

The coexistence of macroscopic fat, enhancing nonlipomatous solid tissue, thick septa, necrotic areas, and infiltrative retroperitoneal extension was highly suggestive of a dedifferentiated liposarcoma rather than a purely well-differentiated lipomatous tumor.

**Fig. 3:**
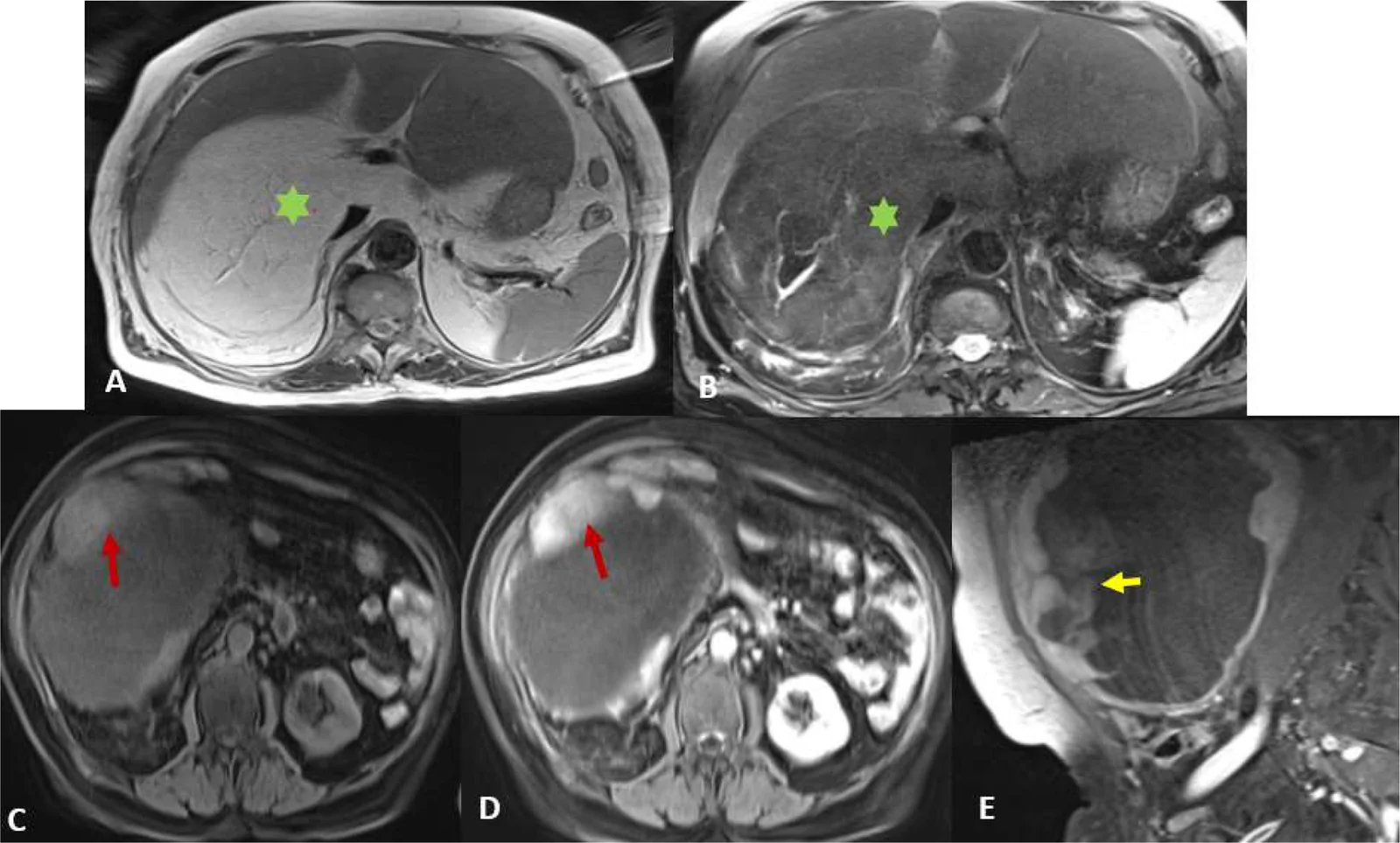
Abdominopelvic MRI, axial T2 weighted sequence without (A) and with fat saturation (B), axial unenhanced T1-weighted sequence (C), axial (D) and sagittal (E) T1 Gadolinium-enhanced sequence showing the fatty portion (green star), thickened septa (yellow arrow), and the enhanced soft tissue portion (red arrow).

For further lesion characterization, multiparametric abdominopelvic MRI was performed. It confirmed a giant retroperitoneal mass with secondary intraperitoneal extension, measuring 200 × 140 × 250 mm. The lesion demonstrated a heterogeneous appearance, consisting of abundant macroscopic fat intermingled with a large nonlipomatous solid component and multiloculated necrotic areas. The macroscopic fat component showed complete signal loss on fat-suppressed sequences, whereas the solid component exhibited intermediate T1 and heterogeneous high T2 signal intensity, with heterogeneous post-contrast enhancement. Diffusion-weighted imaging demonstrated marked diffusion restriction within the enhancing nonlipomatous component, confirmed by corresponding low ADC values, while the necrotic areas showed no enhancement or diffusion restriction. These imaging findings strongly favored the diagnosis of a dedifferentiated liposarcoma (Fig. 3).

**Fig. 4:**
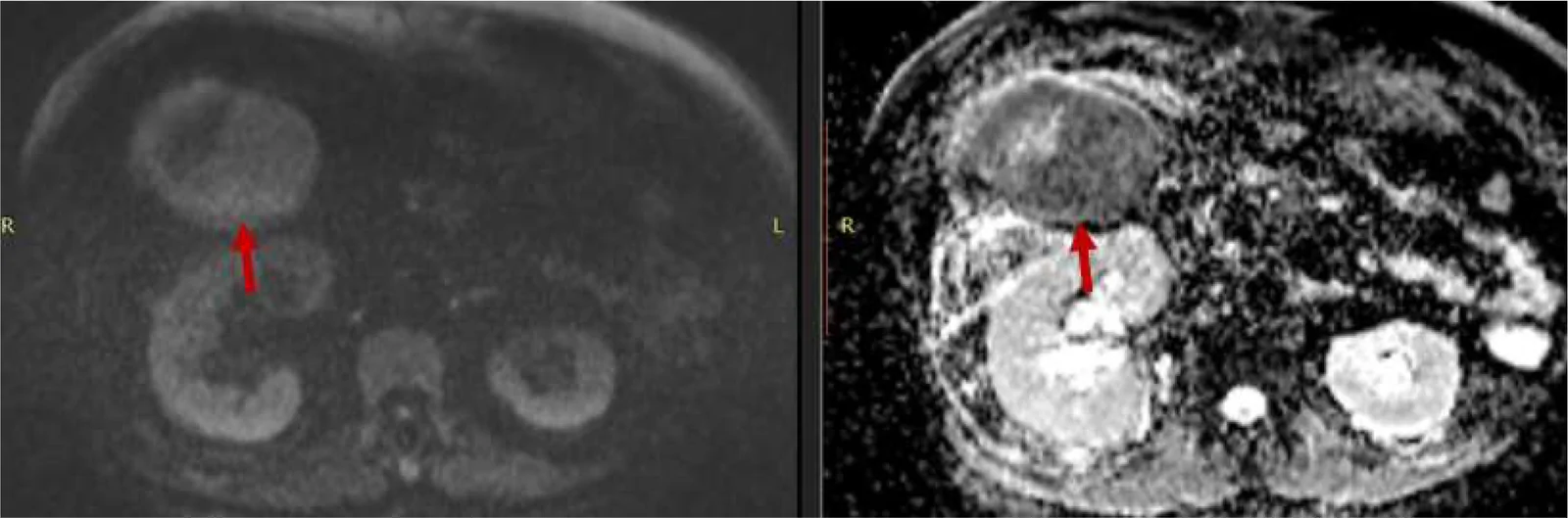
Diffusion-weighted imaging demonstrated marked diffusion restriction within the enhancing nonlipomatous component, confirmed by corresponding low ADC values.

**Fig. 5:**
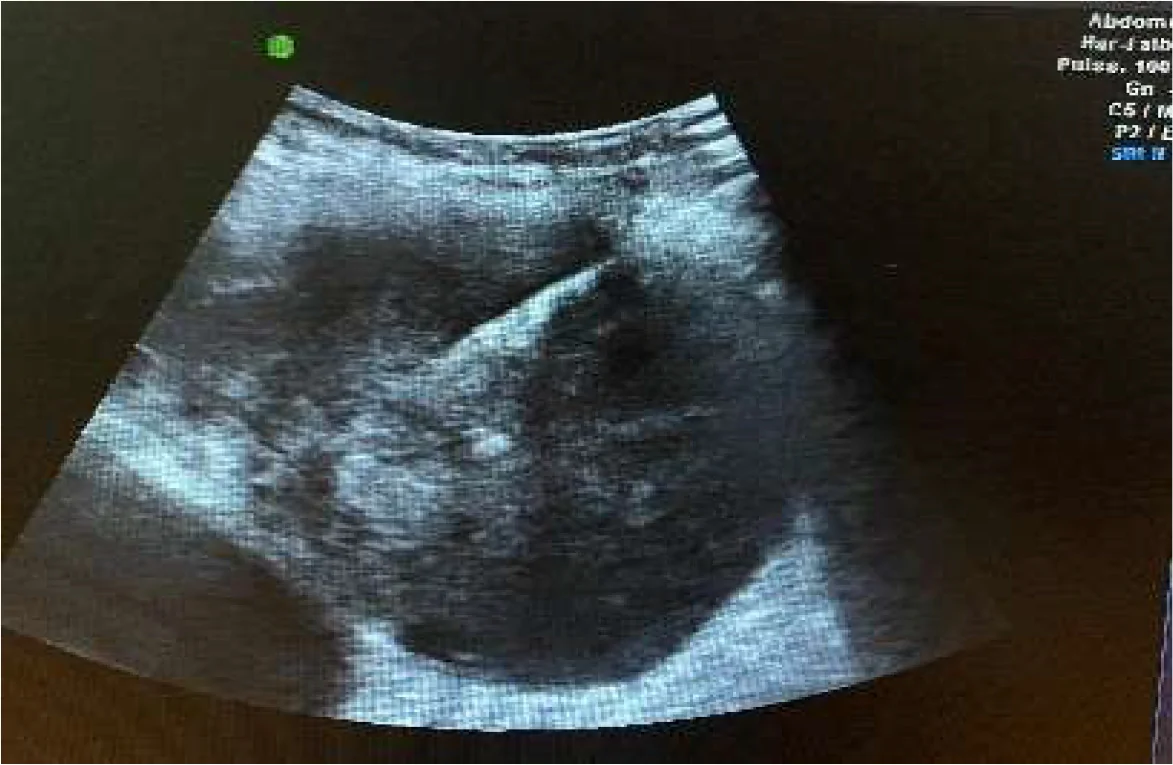
Ultrasound-guided biopsy of the solid component of the tumor using a 14-Gauge semi-automatic needle.

After multidisciplinary consultation, considering the intraperitoneal extension and the contact with critical structures such as the major abdominal vessels, surgery was not indicated. Furthermore, the benefit-risk ratio favored an ultrasound-guided biopsy for histological confirmation, despite the risk of potential dissemination. The patient underwent an ultrasound-guided biopsy of the tissue portion (Fig. 4) and the histopathological examination confirmed the diagnosis of a dedifferentiated liposarcoma (Fig. 5).

## Discussion

Retroperitoneal liposarcoma is one of the most common primary retroperitoneal sarcomas despite its rarity, representing less than 1% of all malignancies. It originates from primitive adipocytic precursor cells exhibiting variable degrees of differentiation and typically occurs in adults between 40 and 60 years of age [1,2].

These tumors usually remain clinically silent until they reach a considerable size, explaining their frequent presentation as giant masses associated with compressive symptoms rather than constitutional manifestations.

Multimodality imaging is essential for the diagnosis and management of retroperitoneal liposarcomas. Beyond defining tumor extent and resectability, it provides characteristic imaging features that help predict the histological subtype and guide biopsy and treatment planning.

### Imaging features of dedifferentiated liposarcoma

In the present case, several imaging findings strongly favored the diagnosis of dedifferentiated liposarcoma. CT demonstrated a giant retroperitoneal mass composed predominantly of macroscopic fat associated with a large enhancing nonlipomatous soft-tissue component, thick irregular septa, central necrotic areas, and infiltrative extension into the adjacent retroperitoneal fat with secondary intraperitoneal extension. Retroperitoneal liposarcomas usually produce a mass effect with displacement of adjacent organs rather than direct invasion, although fascial, vascular, or osseous invasion may occur in advanced disease [3,4]. Therefore, the utility of CT is in providing a global assessment of the tumor, its invasion, and vascular emboli, specifying the extent of any thrombus, and remaining a key tool in staging within the TNM classification [1].

MRI further improved lesion characterization by confirming the fatty component on fat-suppressed sequences and demonstrating heterogeneous enhancement and true diffusion restriction within the nonlipomatous component, confirmed by corresponding low ADC values. These imaging findings reflect increased cellularity and represent characteristic features of dedifferentiated liposarcoma [5,6].

The differential diagnosis of a large fat-containing retroperitoneal mass includes atypical lipomatous tumor/well-differentiated liposarcoma (ALT/WDLPS), renal angiomyolipoma, mature teratoma, adrenal myelolipoma, mesenteric lipodystrophy, and fat necrosis.

ALT/WDLPS generally consists predominantly of mature fat with thin or mildly thickened septa and lacks a substantial enhancing nonlipomatous component, extensive necrosis, or diffusion restriction [7].

Renal angiomyolipoma originates from the renal cortex and can demonstrates marked hypervascularity with enlarged intratumoral vessels and contains very small portion of fat. They are typically highly hyperechoic, equal to the echogenicity of renal sinus fat [8].

Mature teratomas usually contain macroscopic fat component, calcifications (tooth-like structures) and nonlipomatous soft tissue component.

Adrenal myelolipomas are well-circumscribed adrenal lesions composed of fat intermixed with hematopoietic tissue and generally remain much smaller than retroperitoneal liposarcomas [9].

Mesenteric lipodystrophy and fat necrosis represent benign inflammatory processes that do not exhibit aggressive features such as nodular enhancement, infiltrative retroperitoneal growth, or restricted diffusion.

In our patient, the coexistence of abundant macroscopic fat, a large enhancing nonlipomatous component, thick enhancing septa, central necrosis, infiltrative retroperitoneal behavior, and diffusion restriction strongly favored the diagnosis of dedifferentiated liposarcoma before histopathological confirmation.

### Role of MRI and biopsy

Although CT remains the first-line imaging modality for evaluating retroperitoneal sarcomas, MRI provides superior tissue characterization and better delineates the relationship between the tumor and adjacent structures. Fat-suppressed sequences accurately confirm the presence of macroscopic fat, while contrast-enhanced and diffusion-weighted sequences facilitate identification of the most aggressive nonlipomatous component, which represents the optimal target for image-guided core needle biopsy [10].

Histopathological examination remains mandatory for definitive diagnosis and should ideally include immunohistochemistry and assessment of MDM2 amplification whenever available to distinguish dedifferentiated liposarcoma from other lipomatous neoplasms.

### Therapeutic implications

Complete en bloc surgical resection with negative margins remains the cornerstone of treatment whenever technically feasible. In our patient, the lesion was considered unresectable after multidisciplinary evaluation by the institutional sarcoma team. Palliative systemic chemotherapy was selected according to the patient’s clinical status. The management of retroperitoneal liposarcoma should always involve a multidisciplinary team including radiologists, surgeons, pathologists, and medical oncologists to optimize local control while preserving adjacent organs whenever possible.

## Conclusion

This case highlights the pivotal role of multimodality imaging in the preoperative diagnosis of giant retroperitoneal dedifferentiated liposarcoma. The combination of abundant macroscopic fat, a large enhancing nonlipomatous component, thick septa, necrosis, infiltrative retroperitoneal growth, and diffusion restriction strongly suggests dedifferentiation before histopathological confirmation. Early recognition of these characteristic imaging features is essential to guide biopsy, optimize multidisciplinary management, and improve therapeutic decision-making.

## Author contributions

IH: Contributed to conception and design; Contributed to analysis; Drafted the manuscript; Gave final approval; Agrees to be accountable for all aspects of work ensuring integrity and accuracy. SE, SO, and FC: Contributed to conception and design; Contributed to analysis; Agrees to be accountable for all aspects of work ensuring integrity and accuracy. NB: Critically revised the manuscript; Gave final approval; Agrees to be accountable for all aspects of work ensuring integrity and accuracy. YG: Contributed to conception and design; Contributed to analysis; Agrees to be accountable for all aspects of work ensuring integrity and accuracy. NA, LC, and SEH: critically revised the manuscript; Gave final approval; Agrees to be accountable for all aspects of work ensuring integrity and accuracy.

## Patient consent

Written informed consent for publication of this case report and associated images was obtained from the patient.
